# Enhancement of 5-aminolevulinic acid-based fluorescence detection of side population-defined glioma stem cells by iron chelation

**DOI:** 10.1038/srep42070

**Published:** 2017-02-07

**Authors:** Wenqian Wang, Kouichi Tabu, Yuichiro Hagiya, Yuta Sugiyama, Yasuhiro Kokubu, Yoshitaka Murota, Shun-ichiro Ogura, Tetsuya Taga

**Affiliations:** 1 Department of Stem Cell Regulation, Medical Research Institute, Tokyo Medical and Dental University (TMDU), Bunkyo-ku, Tokyo, 1138510, Japan; 2 Graduate School of Bioscience and Biotechnology, Tokyo Institute of Technology, 4259-B102, Nagatsuta-cho, Midori-ku, Yokohama, 2268501, Japan; 3 School of Life Science and Technology, Tokyo Institute of Technology, 4259 B-47, Nagatsuta-cho, Midori-ku, Yokohama, 2268501, Japan

## Abstract

Cancer stem cells (CSCs) are dominantly responsible for tumor progression and chemo/radio-resistance, resulting in tumor recurrence. 5-aminolevulinic acid (ALA) is metabolized to fluorescent protoporphyrin IX (PpIX) specifically in tumor cells, and therefore clinically used as a reagent for photodynamic diagnosis (PDD) and therapy (PDT) of cancers including gliomas. However, it remains to be clarified whether this method could be effective for CSC detection. Here, using flow cytometry-based analysis, we show that side population (SP)-defined C6 glioma CSCs (GSCs) displayed much less 5-ALA-derived PpIX fluorescence than non-GSCs. Among the C6 GSCs, cells with ultralow PpIX fluorescence exhibited dramatically higher tumorigenicity when transplanted into the immune-deficient mouse brain. We further demonstrated that the low PpIX accumulation in the C6 GSCs was enhanced by deferoxamine (DFO)-mediated iron chelation, not by reserpine-mediated inhibition of PpIX-effluxing ABCG2. Finally, we found that the expression level of the gene for heme oxygenase-1 (HO-1), a heme degradation enzyme, was high in C6 GSCs, which was further up-regulated when treated with 5-ALA. Our results provide important new insights into 5-ALA-based PDD of gliomas, particularly photodetection of SP-defined GSCs by iron chelation based on their ALA-PpIX-Heme metabolism.

Tumors often display cellular heterogeneity with a hierarchy starting from self-renewing cancer stem cells (CSCs)^1^,^2^,^3^. CSCs are known to be responsible for tumor initiation and resistance to conventional therapeutic treatments, resulting in recurrence^4^,^5^. Thus, effective detection and elimination of CSCs are critical for complete eradication of cancers. In a number of cancers, the side population (SP) method has been proven to be applicable for the identification of CSCs^6^. Previously we demonstrated that rat C6 glioma cells contain a small population of Hoechst 33342 dye-effluxing SP cells^7^, which was confirmed to fit the criteria of glioma CSCs (GSCs): These SP cells possess higher self-renewal ability, for instance, they could produce both SP and non-SP cells, and also form spheres in the serum-free media with bFGF and PDGF. In addition, they have the potential to differentiate into multiple cell types. Most importantly, SP cells have orthotopically higher tumorigenic activity compared with Hoechst 33342-retaining main population (MP) cells as non-GSCs^8^,^9^.

In recent years, 5-aminolevulinic acid (5-ALA)-based photodynamic diagnosis (PDD) and therapy (PDT) are the cutting edge technologies for detection and treatment of cancers, especially malignant gliomas^10^,^11^,^12^. 5-ALA is a key precursor in the heme biosynthesis pathway and metabolized to an intermediate substance protoporphyrin IX (PpIX) with photosensitizing ability. PpIX is preferentially accumulated in tumor cells after administration of 5-ALA in comparison to their normal counterparts, which provides the basis for the application of 5-ALA-based method in oncology^13^,^14^.

Although 5-ALA has been used in many clinical trials, its widespread applications are limited because of insufficient and heterogeneous PpIX accumulation in cancer cells^15^,^16^. Thus, various therapeutic strategies have been proposed to overcome these limitations, including inhibition of PpIX efflux by the suppression of ATP-binding cassette sub-family G member 2 (ABCG2) transporter^17^,^18^,^19^,^20^, potentiation of PpIX synthesis by increasing the activity of enzymes and transporters that are involved in PpIX synthesis^21^,^22^, and reduction of the PpIX to heme conversion by iron removal or relevant enzyme inhibition^23^,^24^,^25^,^26^.

Recently, clinical studies on 5-ALA-mediated PpIX accumulation in glioblastoma multiforme (GBM) were performed^27^,^28^. However, the relationship between PpIX accumulation and GSCs was still unclear. Moreover, it remains to be fully provided that how we could overcome the heterogeneity of cancerous cells in terms of 5-ALA-mediated fluorescence intensities. Therefore, the accurate evaluation of heterogeneous cancer cells and enhancement of PpIX accumulation in the GSCs need to be explored.

Here, using flow cytometry (FACS)-based analysis, we assessed the levels of 5-ALA-mediated PpIX accumulation in C6 glioma CSCs and non-CSCs, and found that the former exhibits lower PpIX fluorescence intensity, among which cells with the poorer ability of PpIX accumulation are highly tumorigenic. Finally, we propose an improved method for 5-ALA-based fluorescence detection of SP-defined GSCs.

## Results

### C6 glioma cells show cellular heterogeneity of 5-ALA-mediated intracellular PpIX accumulation

**Figure 1:**
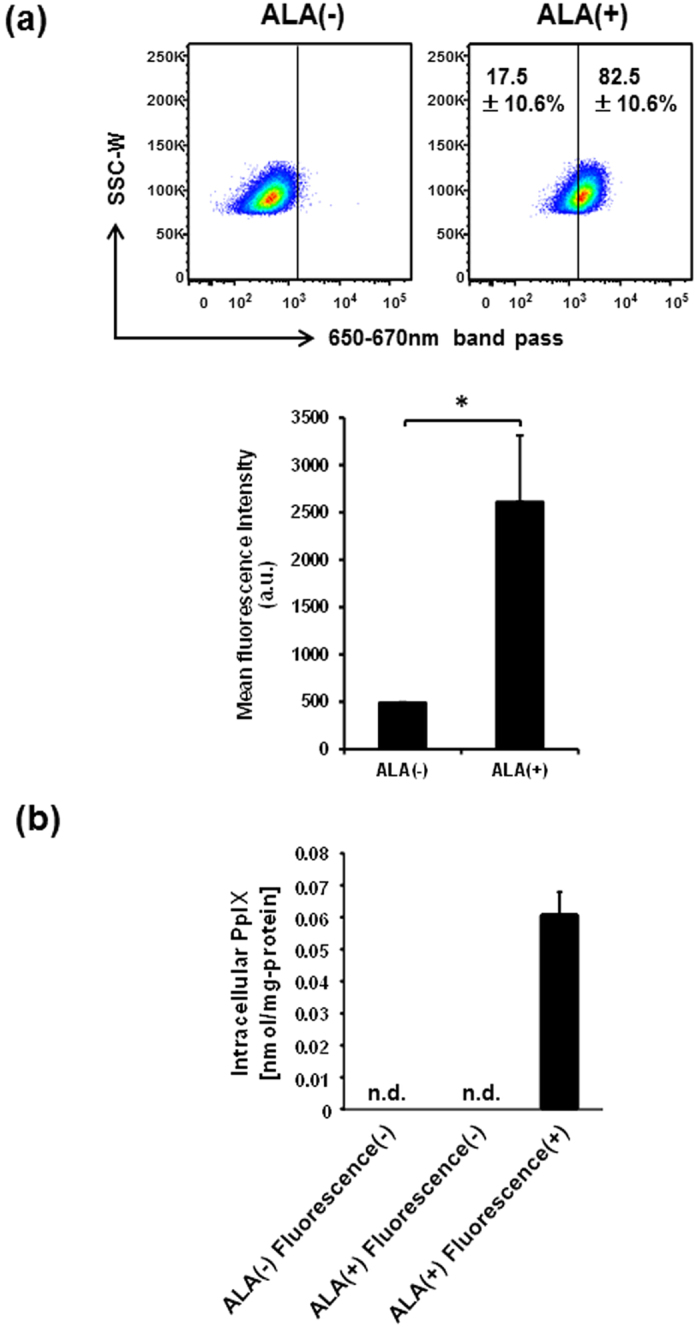
FACS-based detection of intracellular PpIX in C6 glioma cells treated with 5-ALA. (**a**) FACS plots of C6 cells treated and untreated with 5-ALA. The percentages of fluorescence(−) and (+) cells treated with 5-ALA are indicated in representative FACS plots (upper), and mean fluorescence intensities are presented in a bar graph (lower) as means ± SD from three independent experiments. *P < 0.05. (**b**) Detection of intracellular PpIX in 5-ALA-treated C6 cells by HPLC. Fluorescence(−) and (+) cells were sorted from C6 cells treated and untreated with 5-ALA, and analyzed by HPLC. Data are normalized by protein concentrations of lysates and presented in a bar graph as mean ± SD from three independent experiments. n.d., not detected.

To assess the levels of PpIX accumulation in living single cells of C6 glioma, we first treated C6 glioma cells with 5-ALA and analyzed the fluorescence intensity of PpIX by FACS. Fluorescence peak wavelengths of PpIX are known to be observed at 630 and 690 nm with the excitation of 405 and 442 nm^29^. C6 cells were treated with 5-ALA for 4 hours to allow PpIX synthesis and excited with 488 nm laser due to the availability of the lasers equipped on FACS. The emitted fluorescence was detected through a 660/20 nm band-pass filter. The percentage of fluorescence(+) C6 cells and mean fluorescence intensity were significantly increased by 5-ALA treatment (Fig. 1a). Approximately 17.5 ± 10.6% of C6 cells remained at low fluorescence, suggesting that C6 cells have a cellular heterogeneity of 5-ALA-mediated accumulation of fluorescent metabolites.

To further identify the fluorescent metabolites from 5-ALA, PpIX and coproporphyrin III (CPIII) in the lysates of sorted fluorescence(−) and (+) cells were analyzed by high-performance liquid chromatography (HPLC) separation and fluorospectrometer (excitation at 404 nm, detection at 624 nm) analysis^30^. The presence of PpIX was detectable only in the fluorescence(+) cells with 5-ALA treatment but not fluorescence(−) cells with or without 5-ALA treatment (Fig. 1b). CPIII was not detected in all these three groups of sorted cells (data not shown). These results indicate that FACS-based detection of fluorescence is useful to evaluate the levels of 5-ALA-mediated PpIX accumulation in the C6 glioma cell line. These data also suggest that C6 cells are heterogeneous and contain cells with less PpIX accumulation, which may lead to the failure of 5-ALA-based PDD.

### GSCs exhibit less accumulation of PpIX than non-GSCs

**Figure 2:**
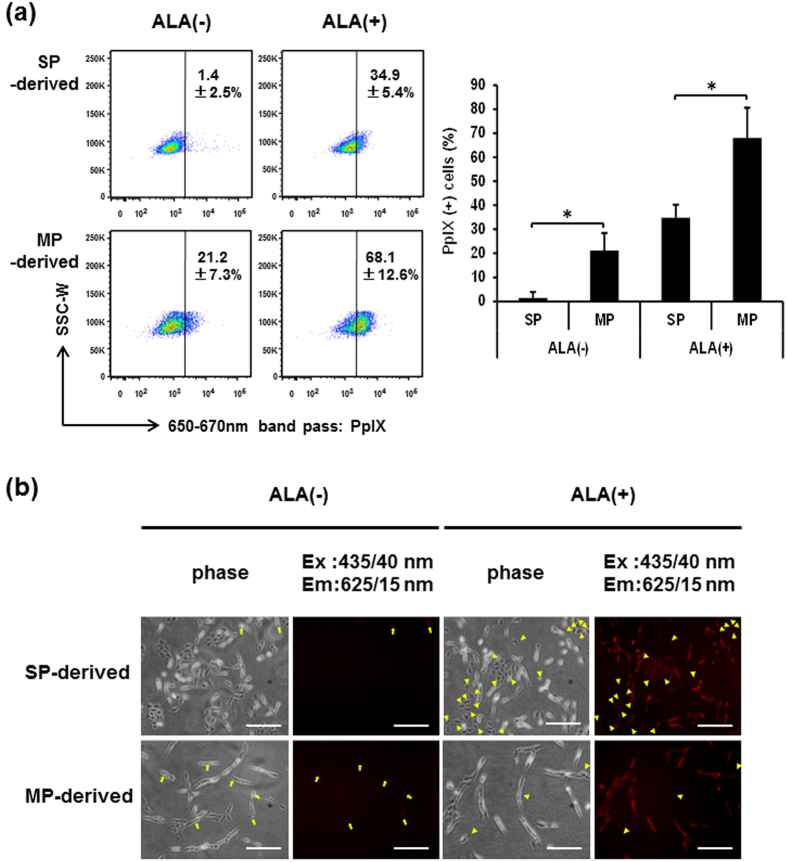
C6 SP-derived cells have significantly less ability of 5-ALA-mediated PpIX accumulation than MP-derived cells. (**a**) FACS plots of C6 SP- and MP-derived cells treated and untreated with 5-ALA. The percentages of PpIX(+) cells are indicated in representative FACS plots (left) and presented in a bar graph (right) as means ± SD from four independent experiments. *P < 0.05. (**b**) Microscopic analysis of PpIX fluorescence in C6 SP- and MP-derived cells treated and untreated with 5-ALA. Representative images from three independent experiments are shown. Arrows: fluorescence(+) cells without 5-ALA. Arrowheads: fluorescence(−) cells with 5-ALA. Scale bars = 200 μm.

To compare the levels of PpIX accumulation between SP-defined GSCs and MP-defined non-GSCs, FACS-sorted C6 SP and MP cells were cultured for 2 days (hereafter “SP-derived cells” and “MP-derived cells”, respectively) and subjected to 5-ALA treatment and flow-cytometric analysis of the intracellular fluorescence corresponding to PpIX (Fig. 2a). Even without 5-ALA treatment, MP-derived cells contain cells with higher level of PpIX fluorescence (21.2 ± 7.3%) than most of the SP-derived cells. More notably, when treated with 5-ALA, 68.1 ± 12.6% of MP-derived cells were positive for PpIX fluorescence, while only 34.9 ± 5.4% of SP-derived cells were positive. It seems that the Hoechst 33342 staining and sorting might somehow affect the 5-ALA-mediated PpIX fluorescence, because more unsorted C6 cells are fluorescent when compared with MP-derived cells as shown in Figs 1a and 2a (82.5 ± 10.6% versus 68.1 ± 12.6%), but the difference is not statistically significant (*P* = 0.17). Taken together, these results suggest that GSCs possess less ability to accumulate PpIX than non-GSCs. To further verify these findings, we next observed the PpIX accumulation under the fluorescence microscope with a QD625 filter cube (excitation band pass filter at 435/40 nm; dichroic mirror with a cut off of 510 nm; emission band pass filter at 625/15 nm) (Fig. 2b). Consistent with the results obtained from FACS analysis using the 488 nm laser and 660/20 band-pass filter, PpIX fluorescence(+) cells were almost negligible in SP-derived cells without 5-ALA treatment (arrows in Fig. 2b, top left two panels). On the other hand, some MP-derived cells showed a faint but visible fluorescence even without 5-ALA treatment (arrows in Fig. 2b, bottom left two panels). When treated with 5-ALA, most MP-derived cells were PpIX fluorescence(+), and the fluorescence(−) cells were very rare under the microscope (arrowheads in Fig. 2b, bottom right two panels). In contrast, more PpIX fluorescence(−) cells were observed in SP-derived cells (arrowheads in Fig. 2b, top right two panels), suggesting that a subset of SP-defined GSCs most likely escapes from detection and resection by 5-ALA-based PDD.

**Figure 3:**
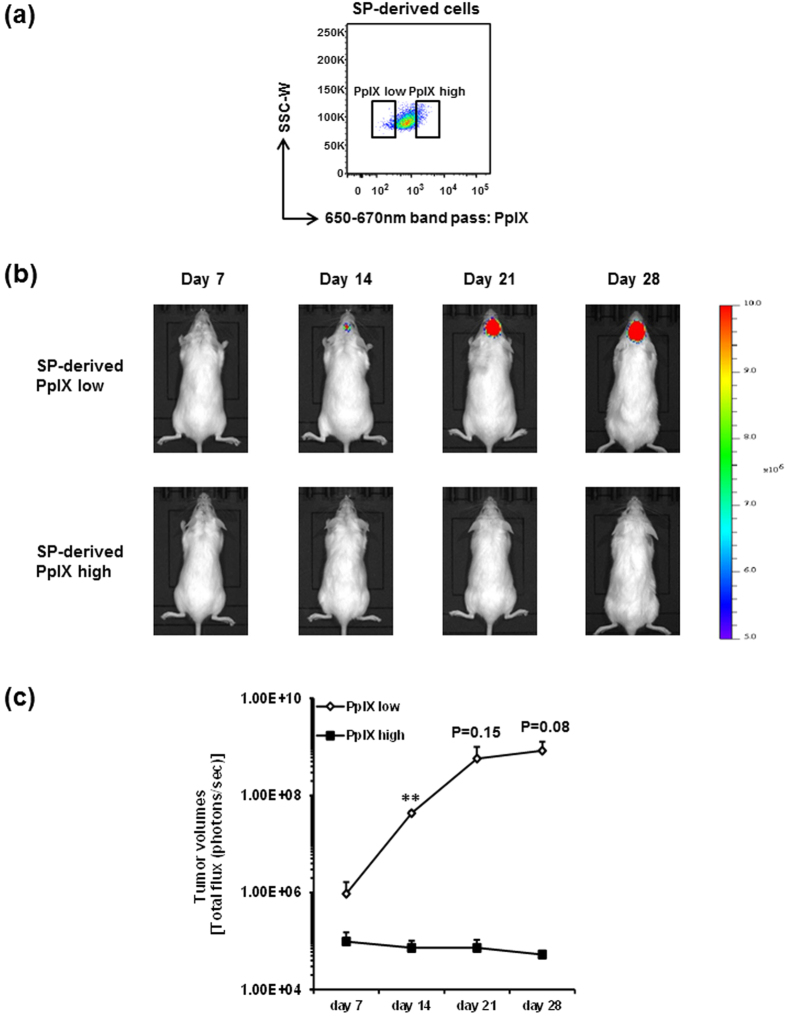
C6 SP-derived PpIX fluorescence low cells possess the tumorigenic capacity. (**a**) Representative FACS plot of PpIX fluorescence low and high cells in C6 SP-derived cells treated with 5-ALA. SP-derived PpIX fluorescence low and high cells were sorted and intracranially transplanted into NOD/SCID mice. (**b**) Representative IVIS images of tumor acquired at day 7, 14, 21 and 28 after transplantation were displayed. Images from other two series of experiments are shown in Supplementary Figure 1. All IVIS images are presented at the same min-max threshold. (**c**) Tumor volumes are assessed by total flux of luminescence signals and shown as means ± SD from three independent experiments. **P < 0.01.

Next, to clarify whether such a subset of escaping GSCs could possess tumorigenicity and eventually lead to cancer recurrence, we assessed the pathological significance of PpIX fluorescence low cell fraction in SP-derived cells. One hundred SP-derived cells with lowest and highest fluorescence of PpIX (Fig. 3a) were intracranially transplanted into the striatum of NOD/SCID mice, and tumor progression was assessed weekly by *in vivo* bioluminescent imaging (IVIS) (Fig. 3b). It is of note that PpIX fluorescence low SP-derived cells exhibited dramatically higher tumorigenic activity and much more rapid progression of tumors than PpIX fluorescence high SP-derived cells in all three independent experiments (Fig. 3b and Supplementary Fig. S1), especially showing a statistically significant increase on day 14 compared to day 7 (Fig. 3c). Surprisingly, PpIX fluorescence high C6 SP-derived cells never form the detectable tumors by IVIS imaging for at least 28 days after transplantation, even though those cells are originated from SP cells. Taken together, these results suggest that SP cells are heterogeneous in their tumorigenicity and that a subset of GSCs possesses an ability to escape from 5-ALA-based PDD, possibly causing failure of resection and leading to cancer recurrence.

### Iron chelation enhances the 5-ALA-mediated PpIX accumulation in GSCs

To obtain clues for developing a strategy to improve 5-ALA-based PDD of SP-defined GSCs for better therapeutic outcome, we next explored the mechanisms of poorer PpIX accumulation in SP cells. As previously described, ABCG2 transporter plays a pivotal role in regulating intracellular PpIX and thereby affecting the efficacy of PDD and PDT^31^,^32^,^33^,^34^. To examine the contribution of ABCG2 to the poor PpIX accumulation in SP cells, SP- and MP-derived cells were treated with 5-ALA in the presence or absence of 10 μM reserpine, a selective ABCG2 inhibitor, and analyzed by FACS for PpIX fluorescence. Reserpine showed no enhancing effect on PpIX accumulation in both SP- and MP-derived cells, but rather decreased the PpIX accumulation in SP-derived cells (Supplementary Fig. S2a), indicating no contribution of ABCG2 transporter to the poor PpIX accumulation in SP-derived cells. When using a different type of inhibitor verapamil that prevents the Hoechst-effluxing ability of SP cells, similar results were obtained regarding the PpIX accumulation (Supplementary Fig. S2b).

**Figure 4:**
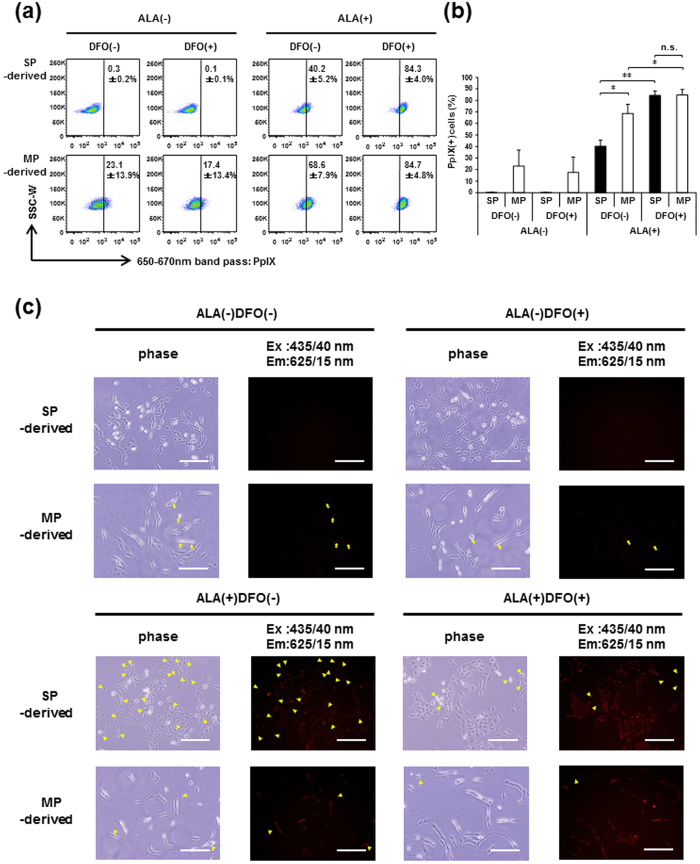
DFO, an iron chelator, increases the accumulation of PpIX in SP-derived cells. Intracellular PpIX accumulation in C6 SP- and MP-derived cells treated with or without 5-ALA in the presence or absence of DFO was analyzed on FACS. The percentages of PpIX(+) cells are indicated in the representative FACS plots (**a**) and presented in a bar graph (**b**) as means ± SD from three independent experiments. **P < 0.01. *P < 0.05. n.s., not significant. (**c**) Microscopic observation of PpIX fluorescence in C6 SP- and MP-derived cells treated with or without 5-ALA in the presence or absence of DFO. Arrows: fluorescence(+) cells without 5-ALA. Arrowheads: fluorescence(−) cells with 5-ALA. Images are representative from three independent experiments. Scale bars = 200 μm.

Because PpIX is metabolized to heme by ferrochelatase (FECH) through ferrous iron incorporation, elimination of iron may suppress this PpIX metabolism process, resulting in the restoration of PpIX accumulation in GSCs. To test this hypothesis, SP- and MP-derived cells were treated with 5-ALA in the presence or absence of an iron chelator, deferoxamine (DFO), and analyzed by FACS for PpIX fluorescence. As shown in Fig. 4a and b, DFO treatment significantly increased the percentage of PpIX fluorescence(+) cells in SP-derived cells to that in MP-derived cells treated with 5-ALA. Microscopic observation of PpIX fluorescence further confirmed that the addition of DFO distinctly decreased the frequency of cells with poor PpIX accumulation (Fig. 4c, arrowheads in bottom 8 panels). These results indicate that iron chelation overcomes the escape of SP-defined GSCs from PDD that otherwise exhibit poor PpIX accumulation.

### Higher expression of heme oxygenase-1 (*HO-1*) gene predicts GBM malignancies

**Figure 5:**
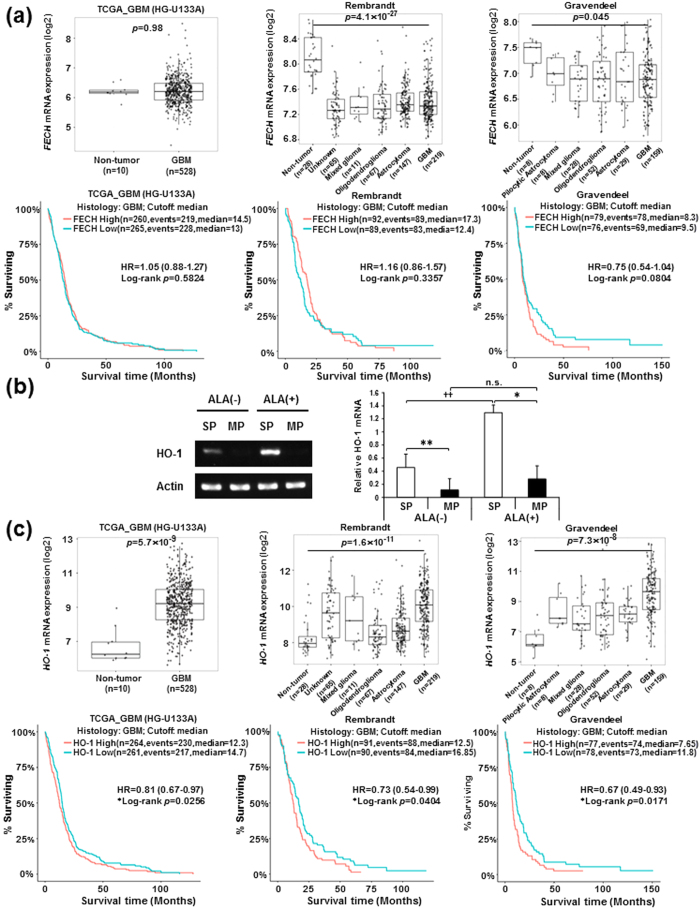
Elevated *HO-1* gene expression is correlated with poor prognosis in GBM patients. (**a**) Box plots for *FECH* mRNA expression in GBM tissues compared with non-tumor tissues (top left panel, TCGA_GBM HG-U133A platform dataset). Box plots for *FECH* mRNA expression in non-tumor tissues and different WHO grades of gliomas (top middle panel, Rembrandt dataset; top right panel, Gravendeel dataset). Data were statistically analyzed by pairwise *t*-tests. Kaplan-Meier survival analysis for human GBM patients with high and low *FECH* expression levels from TCGA_GBM HG-U133A platform, Rembrandt and Gravendeel datasets (Bottom three panels). All observed datasets include IDH-mutated tumors. High and low expression groups are divided by median expression value under statistical analysis with the log-rank test. (**b**) Semi-quantitative RT-PCR analysis for *HO-1* gene expression in SP- and MP-derived cells treated and untreated with 5-ALA. A representative image of gels for the expression of *HO-1* gene is displayed (left). The band intensities of *HO-1* mRNA were normalized by β-actin, and relative *HO-1* mRNA expression was presented in a bar graph (right) as means ± SD from three independent experiments. **P < 0.01, *P < 0.05, between SP and MP cells. ^††^*P* < 0.01 between ALA(−) and (+). n.s., not significant. (**c**) Box plots for *HO-1* mRNA expression in GBM tissues compared with non-tumor tissues (top left panel, TCGA_GBM HG-U133A platform dataset). Box plots for *HO-1* (*HMOX1* in the dataset) mRNA expression in non-tumor tissues and different WHO grades of gliomas (top middle panel, Rembrandt dataset; top right panel, Gravendeel dataset). Data were statistically analyzed by pairwise *t*-tests. Kaplan-Meier survival analysis for human GBM patients with high and low *HO-1* expression levels from TCGA_GBM HG-U133A platform, Rembrandt and Gravendeel datasets (Bottom three panels). All observed datasets include IDH-mutated tumors. High and low expression groups are divided by median expression value under statistical analysis with the log-rank test.

To find the potential cause of poor PpIX accumulation in SP-defined GSCs, we first assessed the relevance of the *FECH* gene, encoding an iron-dependent enzyme that converts PpIX to heme, in human glioma malignancies. We found that there is no significant difference in the *FECH* expression between non-tumor and GBM tissues in the Cancer Genome Atlas (TCGA) dataset (Fig. 5a, top left panel), and its expression is significantly lower in GBM tissues compared with non-tumor tissues in the Repository for Molecular Brain Neoplasia Database (REMBRANDT) and Gravendeel datasets (Fig. 5a, top right two panels). Also, no distinct difference was observed between the expression of *FECH* in GBMs versus lower grades of gliomas (Supplementary Fig. S5). Furthermore, analysis of patients’ survival showed that *FECH* expression is not correlated with the prognosis of GBM patients in all examined datasets (Fig. 5a, bottom three panels). Therefore, we next focused on *HO-1* gene, which encodes a rate-limiting enzyme for heme degradation and functions as an inducible protective gene against cellular stress and oxidative injury^35^. Previously we have already identified *HO-1* in cDNA microarray analysis (available in NCBI GEO repository, #GSE72431) as a gene that is specifically up-regulated in SP cells (Supplementary Table S1)^8^,^9^. In semi-quantitative RT-PCR analysis, even without 5-ALA treatment, SP-derived cells exhibited significantly higher expression of *HO-1* gene than MP-derived cells, and this difference of expression was dramatically increased by 5-ALA treatment (Fig. 5b). These results suggest that higher expression levels of *HO-1* may accelerate the PpIX/heme metabolic pathway, leading to the poor 5-ALA-mediated accumulation of PpIX in SP-defined GSCs. Finally, to further examine the clinical significance of HO-1, we used publicly available datasets and found that *HO-1* (*HMOX1* in the dataset) expression is significantly elevated in GBM tissues compared with non-tumor tissues in all tested datasets (Fig. 5c, top three panels). Supportively, *HO-1* expression is significantly higher in WHO grade IV (most malignant) GBMs in all datasets when compared with lower grades of gliomas (Supplementary Fig. S6). Also, higher *HO-1* (*HMOX1* in the dataset) expression was found to be significantly correlated with poorer prognosis in GBM patients, although its difference is not dramatic (Fig. 5c, bottom three panels). In lower grade gliomas (astrocytomas and oligodendrogliomas), patients with higher expression of *HO-1* gene evidently exhibit poorer survival (Supplementary Fig. S7). These data indicate that *HO-1* gene expression is associated with human glioma malignancies.

## Discussion

**Figure 6:**
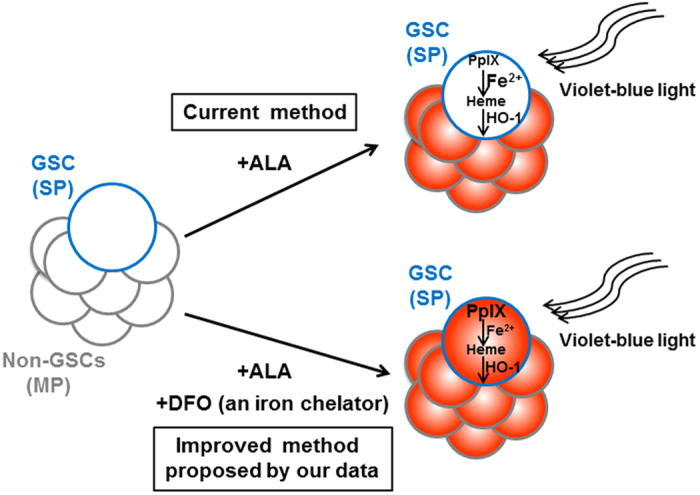
A schematic summary of improved method for 5-ALA-based PDD of SP-defined GSCs. SP-defined GSCs exhibit lower PpIX accumulation in 5-ALA-based PDD, potentially due to accelerated iron usage to convert PpIX to heme and the increased expression of HO-1 to accelerate PpIX-heme metabolism. The combined use of an iron chelator DFO dramatically restores the decreased accumulation of PpIX in SP-defined GSCs.

Chemo/radio-therapy after surgical resection could dispose of most cancer cells, but CSCs likely survive such therapies, which results in cancer recurrence. Thus, detection of residual CSCs during surgery is a promising strategy for cancer eradication, but little has been investigated. In this study, we show that in at least rat C6 glioma, SP-defined GSCs have an ability to escape from 5-ALA-based photodynamic detection due to significantly lower accumulation of PpIX, and finally propose that iron chelation could overcome it (Fig. 6).

Interestingly, even without 5-ALA treatment, a portion of MP-derived cells displays PpIX fluorescence which is negligible in SP-derived cells (Fig. 2). 5-ALA is naturally synthesized from glycine and succinyl-coenzyme A in the mitochondria by ALA synthase (ALAS). Because the expression of ALAS1 gene, the ubiquitous form of ALAS, is relatively high in both SP and MP cells (Supplementary Table. S1), C6 glioma cells may endogenously synthesize ALA and MP cells could accumulate steady state level of PpIX due to slower conversion of PpIX to heme.

We performed xenografts of the PpIX fluorescence lowest and highest groups among SP cells, and only the former exhibited tumorigenic activity in NOD/SCID mouse brain (Fig. 3). To examine whether PpIX accumulation could affect cell survival, PpIX fluorescence low and high cells from cultured SP cells were sorted after treatment with 5-ALA for 4 hours, and assessed by trypan blue assay. As shown in Supplementary Fig. S3a, cell viability was comparable between SP-derived PpIX fluorescence low and high cells, demonstrating that there was no difference of cell viability, at least when transplanted. When cultured, PpIX fluorescence high cells exhibited slightly slower cell proliferation on day 4, which is statistically significant, but there is no difference at least during the 2-day culture (Supplementary Fig. S3b,c). Thus, there seems to be some effect of PpIX accumulation on cell proliferative capacity. However, this moderate difference could not fully explain dramatic differences of tumor volumes in transplantation.

Although we did not transplant the PpIX fluorescence intermediate group of SP cells, we have previously showed that SP cells are highly tumorigenic than MP cells when total SP fraction was compared with MP fraction^8^. Thus, PpIX fluorescence highest SP cells are more like MP cells. It could be assumed that SP cells with lower PpIX accumulation that are more highly tumorigenic may escape from 5-ALA-based PDD and induce the recurrence of tumors.

Our data imply that PpIX fluorescence high cells are non-tumorigenic. When we treated C6 glioma cells with 1 mM 5-ALA for 4 hours, and PpIX fluorescence low and high cells were stained with Hoechst 33342 immediately after sorting (Supplementary Fig. S4a), SP percentage in the PpIX fluorescence low cells was significantly higher than that in the PpIX fluorescence high cells, but some SP cells exist in the latter (Supplementary Fig. S4b). Considering that PpIX fluorescence high C6 SP cells showed non-tumorigenic ability (Fig. 3), these data suggest that C6 SP cells and PpIX fluorescence low/negative cells are heterogeneous in their tumorigenicity. Taken together, lack of the PpIX signal is more commonly observed in GSCs, and combination of the SP method and the 5-ALA-staining method should more precisely identify the GSC population. This issue will be further investigated.

Next, inhibition of ABC transporters in SP cells by reserpine or verapamil did not enhance but rather slightly decreased PpIX accumulation (Supplementary Fig. S2), which suggests complex mechanisms: ABCB6 in the mitochondrial membrane likely translocates coprophyrinogen III from cytoplasm into mitochondria and contributes to PpIX synthesis^36^. Reserpine and verapamil might have the affinity to ABCB6 to compete with coprophyrinogen III for its transport into mitochondria and decrease the PpIX synthesis^37^. Other ABC transporters, like ABCB7 and ABCB10, also play pivotal roles in PpIX accumulation by effluxing iron from mitochondria^36^. Inhibition of these transporters might increase amounts of iron in mitochondria, and enhance the conversion of PpIX to heme.

To our knowledge, this is the first report to propose that an iron chelator DFO contributes to the enhancement of PpIX accumulation in SP-defined GSCs. DFO is an iron-specific chelator that has higher binding affinity to ferric iron, and therefore has been used in the treatment of acute and chronic iron overload diseases^38^. Compared with other iron chelators ethylene diamine tetra-acetic acid (EDTA) and a hydroxypyridinone iron chelator 1, 2-diethyl-3-hydroxypyridin-4-one hydrochloride (CP94), DFO was selected in our study as a combinational drug for 5-ALA-based detection of SP-defined GSCs, because DFO is the only iron chelating drug approved by the Food and Drug Administration (FDA) and has been used in clinical treatment for over a period of time with no significant side effects^39^,^40^. Thus, we believe that this improved method could quickly be translated into clinical application.

Studies on the enhancements of 5-ALA-mediated PpIX accumulation for PDD of gliomas have also been conducted by some research groups. Previously, Teng *et al*. demonstrated that silencing of FECH by small interference RNA enhances 5-ALA-mediated PpIX fluorescence in glioma cell lines expressing relatively high levels of FECH^41^. In addition, Zhao *et al*. showed up-regulation of ABCB6 expression in human gliomas compared with normal brain tissues and found that ABCB6 overexpression increases accumulation of PpIX fluorescence in gliomas^42^. Moreover, Valdes *et al*. showed that DFO-mediated iron chelation increases 5-ALA-mediated PpIX accumulation in U251 malignant glioma cells *in vivo*^43^. However, CSCs have never been taken into account in these studies.

Also, Kim JE *et al*. shown that mutational status of isocitrate dehydrogenase 1 (IDH1) is associated with enhanced 5-ALA fluorescence^44^, suggesting that the mechanisms of PpIX accumulation might be diverse depending on the subtypes of gliomas. Previously we have reported that rat C6 glioma cell line represents a model of classical human glioblastoma mutiforme^8^. It remains to be clarified whether the use of an iron chelator DFO in the present study can be applied to all subtypes of gliomas.

In addition to the case of glioma cells, DFO was also found to increase 5-ALA-mediated PpIX accumulation in various tumor cells^45^,^46^,^47^. However, in a clinical study of 8 patients, DFO showed no enhancement of PpIX in superficial basal cell carcinomas or Bowen’s disease, but clear enhancement in normal skin cells^48^. This raises the question whether DFO might increase PpIX accumulation in normal astrocytes during 5-ALA-based PDD of gliomas. However, previous study revealed that there is no 5-ALA-mediated PpIX fluorescence observed in the normal astrocytes isolated from Wistar rat^49^, which is the same source of C6 glioma cell line^50^. Moreover, Valdes *et al*. demonstrated that DFO leads to an increase in PpIX fluorescence in tumors *in vivo* but its effect in normal brain cells was not observed^43^. Further *in vivo* studies are required to test the applicability of DFO in the enhancement of GSCs.

In our study, the complete mechanisms of DFO in the enhancement of PpIX accumulation in SP-defined GSCs have not yet been revealed. Iron is the substrate for conversion of PpIX to heme, and Hayashi *et al*. found that uptake abilities of iron into mitochondria play an important role in tumor-selective PpIX accumulation^51^. Therefore, reduced amounts of iron in mitochondria by DFO may lead to enhanced PpIX accumulation in SP-defined GSCs. On the other hand, Schonberg *et al*. showed that transferrin receptor and ferritin, two core iron regulators, are necessary for GSC tumorigenesis^52^. As iron is required for not only heme biosynthesis pathway but also a wide variety of important cellular functions^53^, it could be conceivable that GSCs preferentially use iron for the maintenance of stemness properties rather than heme synthesis.

Previous studies demonstrated that high expression of HO-1 protects tumor cells from PDT, and inhibition of its enzymatic activity by siRNA or a pharmacological inhibitor could enhance the cytotoxic effects of PDT in colon adenocarcinoma and melanoma cells^54^,^55^. Given that PDT is one of the promising antitumor treatments by reactive oxygen species (ROS) generation^56^, high HO-1 expression in GSCs is involved in an important defense mechanism of oxidative stress. Therefore, HO-1 enzyme could be a reasonable target to enhance 5-ALA-based PDD and PDT in GSCs. Further examination with an iron chelator and HO-1 targeting in PDD and PDT could establish the novel therapeutic strategies to eradicate GSCs.

Our data provide an important implication for using 5-ALA-based PDD to detect GSCs present in glioma patients. For instance, it is currently not possible to surgically resect cancer cells, particularly CSCs, in the invasive front, where the presence of GSCs has been reported^57^. We hope that further clinically oriented investigation of 5-ALA-based PDT in combination with an iron chelator will facilitate the elimination of invasive GSCs for eradication of cancer.

In conclusion, our study demonstrates lower PpIX accumulation in SP-defined GSCs for resisting to detection and proposes that an iron chelator DFO may well be used to improve 5-ALA-based PDD of SP-defined GSCs.

## Methods

### Reagents

5-ALA hydrochloride was purchased from Cosmo Oil Co., Ltd. (Tokyo, Japan). An iron chelator DFO was purchased from Sigma (St Louis, MO, USA).

### Cell culture and SP isolation

The rat C6 glioma cell line was cultured in Dulbecco’s modified Eagle’s medium (DMEM) with 10% fetal bovine serum (FBS) (Sigma, USA). C6 cells stably expressing engineered firefly luciferase (luc2) gene were previously established^8^. SP cells were isolated from the C6-*luc2* cell line by using Hoechst 33342 and flow cytometer as previously described^7^,^8^. All the recombinant DNA experiments in this manuscript were carried out under the approval of Recombinant DNA Experiment Safety Committee of Tokyo Medical and Dental University (2013–086 A).

### 5-ALA treatment and HPLC analysis of porphyrins

The rat C6 glioma cells were incubated with 1 mM 5-ALA for 4 hours. For HPLC analysis, 2 × 10^5^ PpIX fluorescence(−) and (+) cells were sorted from C6 cells treated and untreated with 5-ALA, rinsed and lysed with 0.1 N NaOH. Aliquots of cell lysates were used for protein concentration measurement (Bio-Rad Protein Assay Dye Reagent Concentrate, Bio-Rad Laboratories, Inc., CA, USA), whereas the remaining 50 μl of lysates were denatured by addition of 3-fold volume (150 μl) of N, N- dimethylformamide: isopropanol (100:1, v/v). The samples were vortexed and centrifuged at 14000 rpm for 10 min at 4 °C, and the collected supernatants were subjected to HPLC analysis after overnight storage in the dark as previously described^49^. Briefly, porphyrins were separated by the HPLC system (Prominence, Shimadzu, Kyoto, Japan) equipped with a reversed-phase C_18_ column (CAPCELL PAK, C18, SG300, 5 μm, 4.6 mm × 250 mm, Shiseido Co., Ltd., Tokyo, Japan). Elution solvents were solvent A (1 M ammonium acetate including 12.5% acetonitrile, pH 5.2) and solvent B (50 mM ammonium acetate including 80% acetonitrile, pH 5.2). Elution was performed with solvent A for 5 min and subsequently with a linear gradient of solvent B (0–100%), followed by elution with solvent B for 10 min. The elution flow was constant at a rate of 1.0 ml/min. Porphyrins were continuously detected using a spectrophotometer (excitation at 404 nm, detection at 620 nm). The concentrations of porphyrins were estimated from calibration curves of reference standards.

### FACS analysis and microscopic observation of PpIX fluorescence

The fluorescence intensity of cellular PpIX was determined by FACS and fluorescence microscopy. After SP and MP cells were sorted and re-cultured for 2 days, media were changed to fresh media containing 1 mM 5-ALA and cells were additionally incubated for 4 hours. In some cases, DFO (100 μM) was added into media together with 5-ALA. PpIX fluorescence was measured with a FACS Aria II (BD Biosciences) using 488 nm laser and a 660/20 nm band-pass filter and fluorescence microscope (BioRevo BZ-9000, Keyence, Japan) equipped with a QD625 filter cube (Olympus, Japan).

### Semi-quantitative RT-PCR analysis

Total RNAs were extracted from SP and MP cells using ISOGEN (WAKO). First-strand cDNA was synthesized from 2 μg total RNA using SuperScript III First-Strand Synthesis system for RT-PCR kit (Invitrogen). PCR reactions were performed using KOD Plus (TOYOBO, Japan) according to the manufacturer’s instruction. PCR primer sequences are as follows: rat *HO-1*, 5′**-**AAGAGGCTAAGACCGCCTTC-3′ (forward), 5′-GCATAAATTCCCACTGCCA-3′ (reverse); rat *Actb*, 5′-ACCAGGGTGTGATGGTGGG-3′ (forward), 5′-CAGCCTGGATGGCTACGTACA -3′ (reverse). After agarose gel electrophoresis, the density of PCR products for *HO-1* gene was calculated using ImageJ software (1.48 v), and normalized by that for *Actb* gene.

### Tumor transplantation and IVIS imaging

One hundred PpIX fluorescence low and high cells were sorted from SP-*luc2* by FACS, and intracranially transplanted into the right striatum of NOD.CB17-*Prkdc*^*scid*^/J mice (female, 6–12 weeks of age, Charles River Japan, Yokohama, Japan). For assessment of tumor volumes by IVIS, transplanted mice were intraperitoneally injected with d-luciferin (150 mg/kg in PBS; Summit Pharmaceuticals International Corp). After 15 minutes, mice were placed in a dark imaging chamber under isoflurane anesthesia, and imaged with the Xenogen IVIS Lumina system (binning, high; f/stop, 1; field-of-view, 16.5 mm × 16.5 mm). The exposure times were adjusted so that the luminescence does not saturate. Data analysis was performed using Living Image v2.5 software (Xenogen). In radiance mode, fixed size of regions of interest (ROIs) were drawn covering the whole tumor in each mouse. The luminescence signals (total flux; photons/sec) were quantitated by subtracting background fluorescence from tumor signal. Animal procedures were conducted according to the protocols approved by Animal Care and Use Committee of TMDU (authorization number, 0106208A and 0170281A).

### Bioinformatic analysis

Target gene expression between non-tumor and GBM tissues and patients’ clinical outcome were analyzed in TCGA_GBM (HG-U133A), Rembrandt and Gravendeel datasets by using GlioVis (http://gliovis.bioinfo.cnio.es/).

### Statistical analysis

All comparisons between experimental groups, except for bioinformatics analysis, were made by Student’s *t*-test. Target gene expression between non-tumor and GBM tissues was tested by pairwise *t* tests with corrections for multiple testing (*p* values with Bonferroni correction). For Kaplan-Meier survival curves, *p* values were obtained by using the log-rank test.

## Additional Information

**How to cite this article**: Wang, W. *et al*. Enhancement of 5-aminolevulinic acid-based fluorescence detection of side population-defined glioma stem cells by iron chelation. *Sci. Rep.* **7**, 42070; doi: 10.1038/srep42070 (2017).

**Publisher's note:** Springer Nature remains neutral with regard to jurisdictional claims in published maps and institutional affiliations.

## Supplementary Material

Supplementary Information
