# Supplementary material for: Enhancement of 5-aminolevulinic acid-based fluorescence detection of side population-defined glioma stem cells by iron chelation
Source: Sci Rep. 2017 Feb 7;7:42070. doi: 10.1038/srep42070 (PMC5294410; doi:10.1038/srep42070)
Supplement: Supplementary Information [file srep42070-s1.pdf]

Supplementary Information

Enhancement of 5-aminolevulinic acid-based fluorescence detection of side population-defined glioma stem cells by iron chelation

Wenqian Wang[1], Kouichi Tabu[1§], Yuichiro Hagiya[2], Yuta Sugiyama[2], Yasuhiro Kokubu[1], Yoshitaka Murota[1], Shun-ichiro Ogura[2], Tetsuya Taga[1§]

[1]Department of Stem Cell Regulation, Medical Research Institute, Tokyo Medical and Dental University (TMDU), Bunkyo-ku, Tokyo, 1138510, Japan

[2]Graduate School of Bioscience and Biotechnology, Tokyo Institute of Technology, 4259-B102, Nagatsuta-cho, Midori-ku, Yokohama, 2268501, Japan

Supplementary Figures S1-S7

Supplementary Table S1

Supplementary Methods

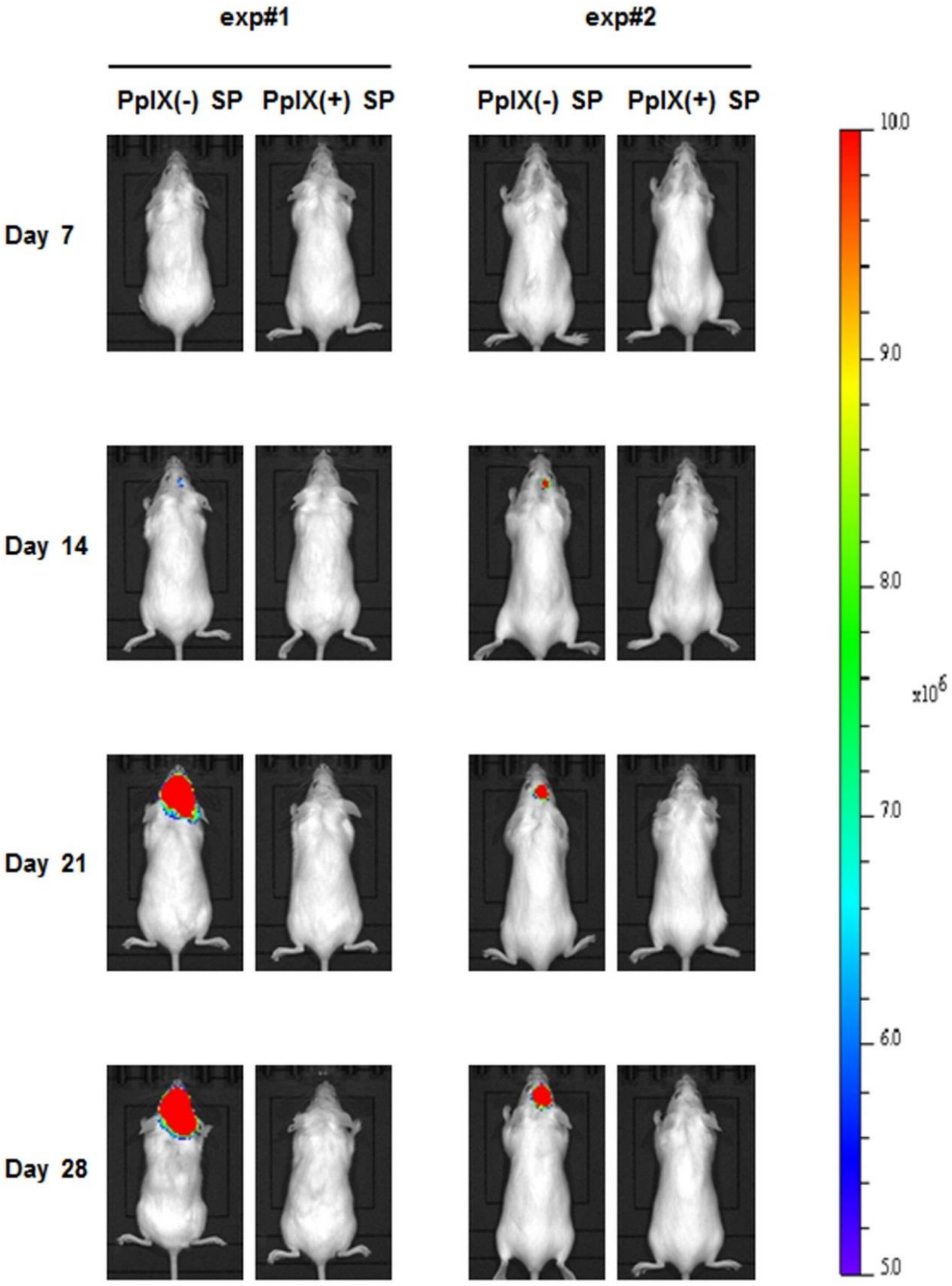

Supplementary Figure S1. C6 SP-derived PpIX fluorescence low cells have higher tumorigenicity (related to Figure 3)

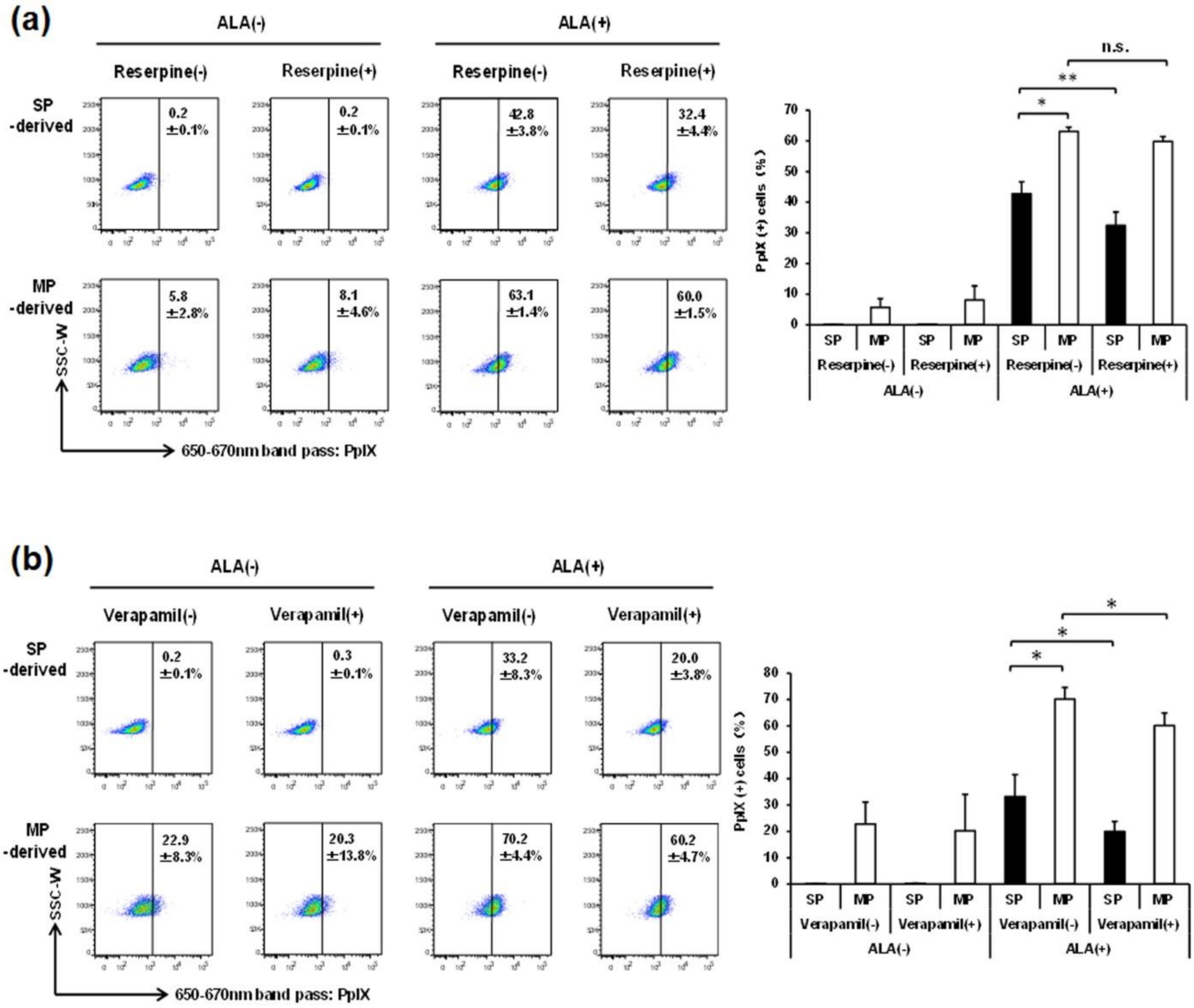

Supplementary Figure S2. Reserpine and verapamil, inhibitors of ABC transporters, have no effects on low accumulation of PpIX in SP-derived cells

Intracellular PpIX accumulation in C6 SP- and MP-derived cells treated with or without 5-ALA in the presence or absence of reserpine (a) or verapamil (b). The percentages of PpIX(+) cells are indicated in representative FACS plots (left) and presented in a bar graph (right) as means±SD from three independent experiments. ***P*<0.01.**P*<0.05.   n.s., not significant.

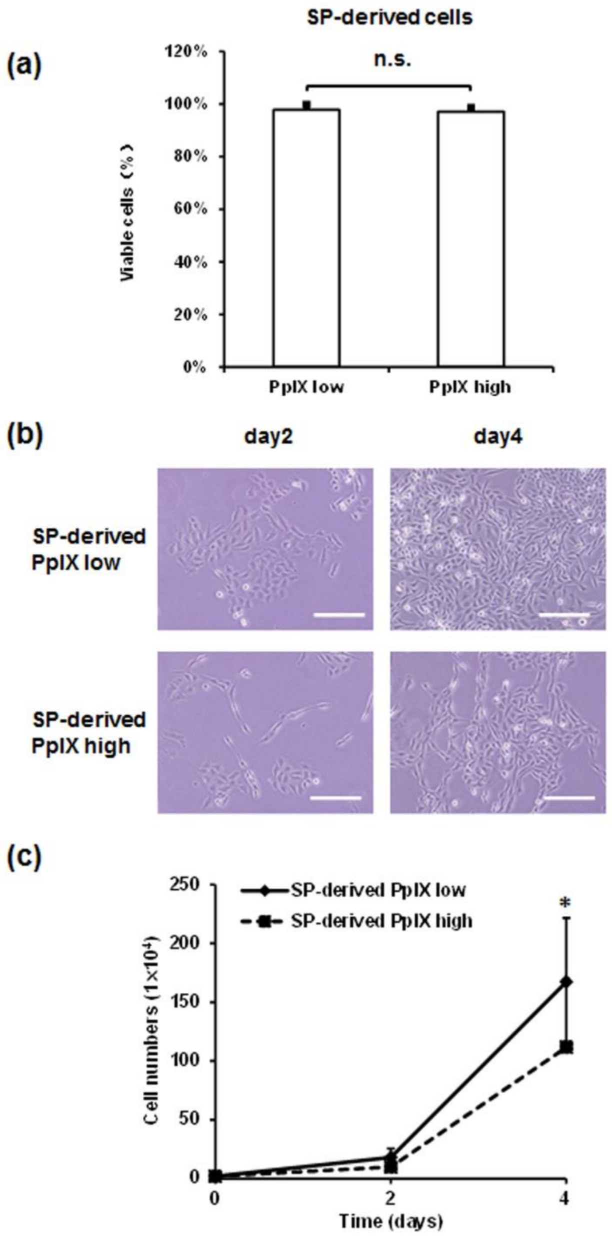

Supplementary Figure S3. Cell viability and growth of SP-derived PpIX fluorescence low and high cells in vitro

(a) The percentages of viable cells in PpIX fluorescence low and high fractions are presented in a bar graph as means±SD from three independent experiments. n.s., not significant. (b) Representative images of SP-derived PpIX fluorescence low and high cells are shown on day 2 and 4 after reculturing. Scale bars, 200 μm. (c) The growth curves of SP-derived PpIX fluorescence low and high cells. Data were presented as means±SD from three independent experiments. **P*<0.05.

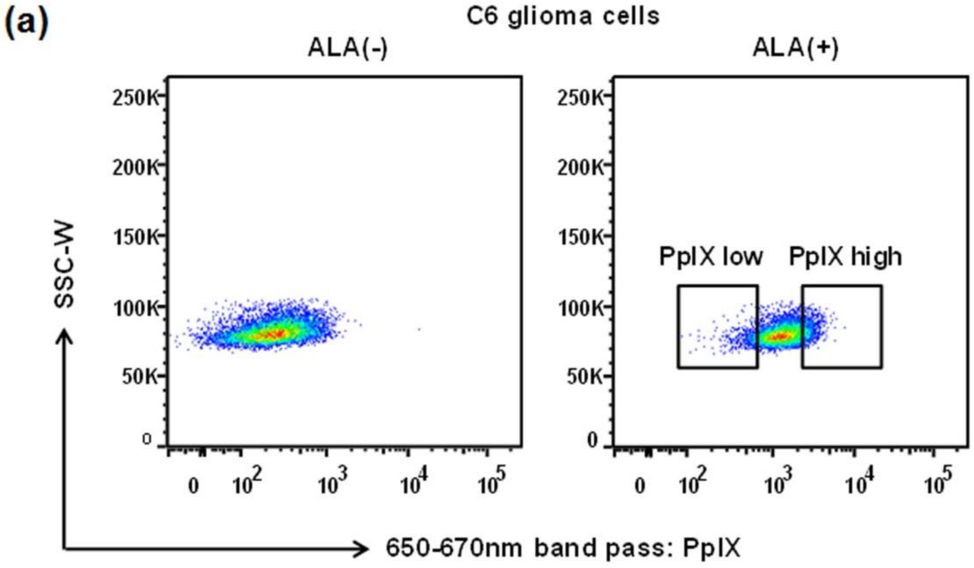

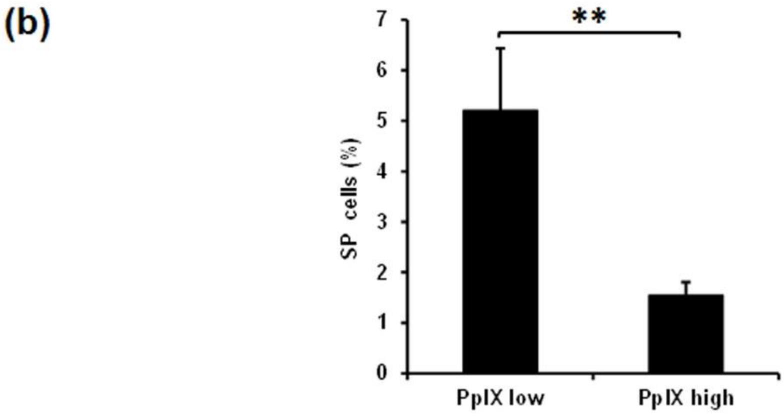

Supplementary Figure S4. C6 glioma PpIX fluorescence low cells contain higher percentage of SP cells

(a) FACS plots of PpIX fluorescence low and high cells in C6 glioma cells treated with 5-ALA. (b) The percentages of SP cells in PpIX fluorescence low and high fractions are presented in a bar graph as means±SD from five independent experiments. ***P*<0.01.

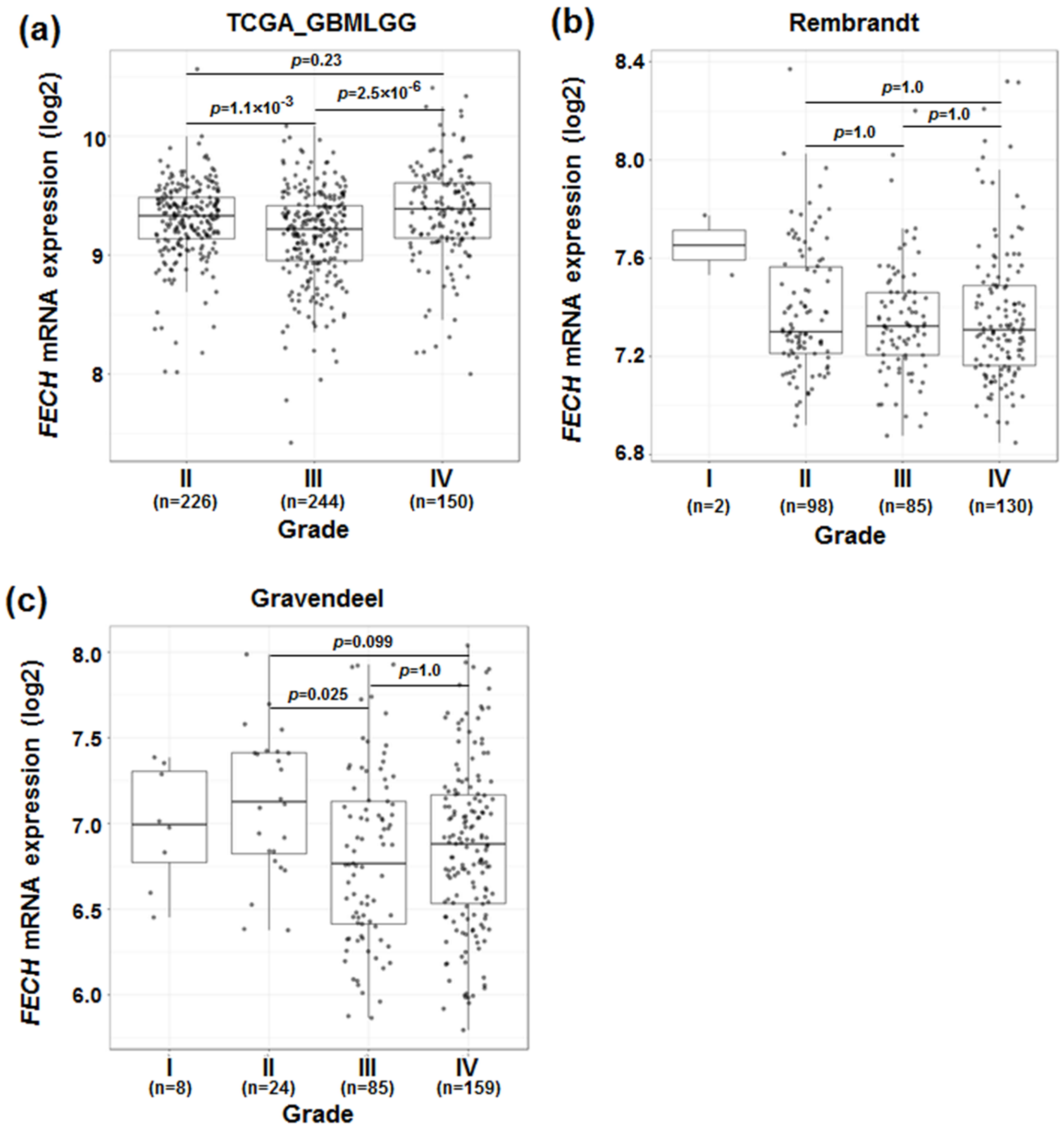

Supplementary Figure S5. ***FECH*** **expression between different grades of human gliomas**

Box plots for *FECH* mRNA expression in different grades of gliomas from TCGA_GBMLGG (a), Rembrandt (b) and Gravendeel (c) datasets are presented. Data were statistically analyzed by pairwise *t*-tests.

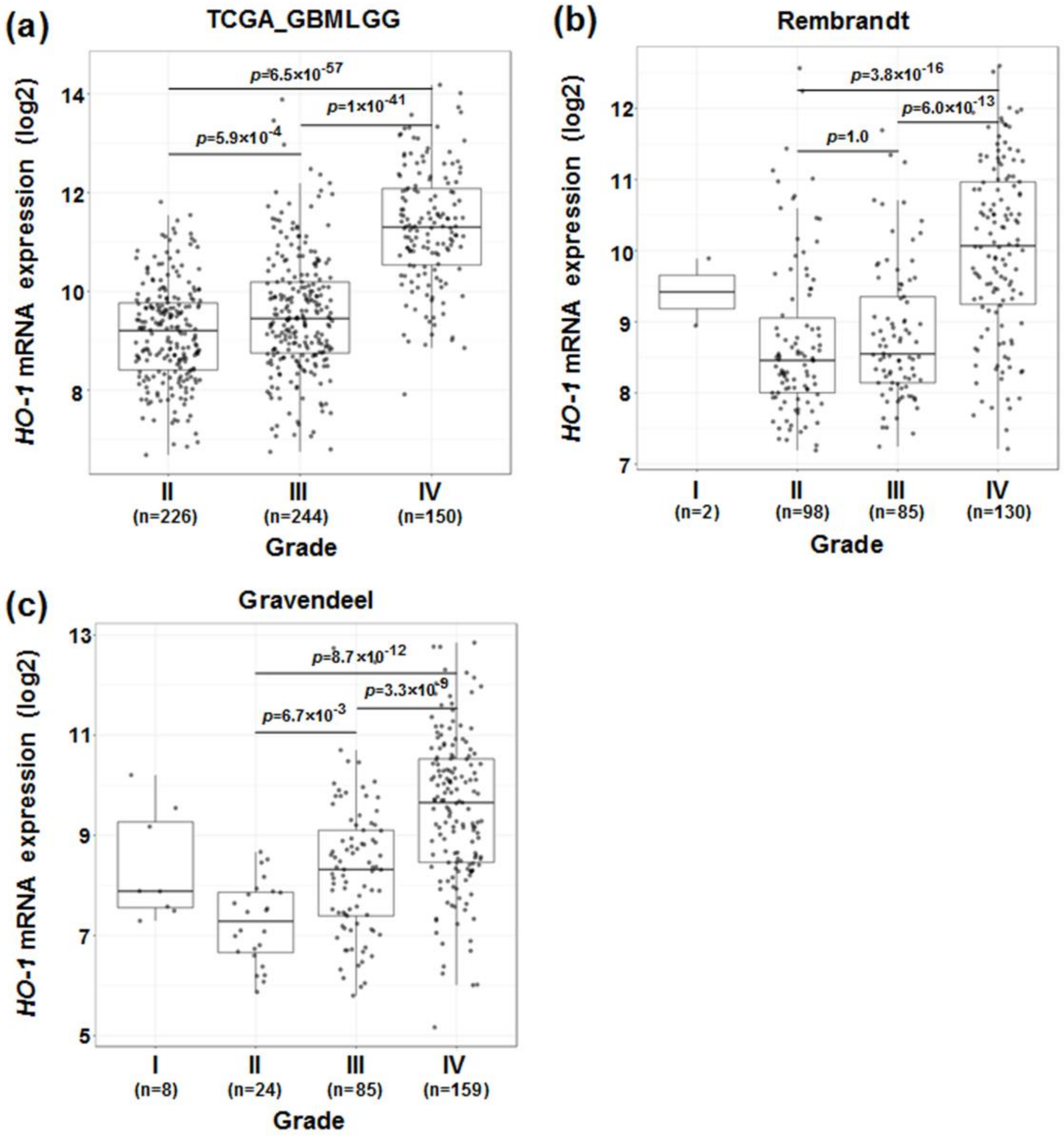

Supplementary Figure S6. ***HO-1*** **expression is higher in WHO grade IV GBMs than in lower grades of gliomas**

Box plots for *HO-1* (*HMOX1* in the dataset) mRNA expression in different grades of gliomas from TCGA_GBMLGG (a), Rembrandt (b) and Gravendeel (c) datasets are presented. Data were statistically analyzed by pairwise *t*-tests.

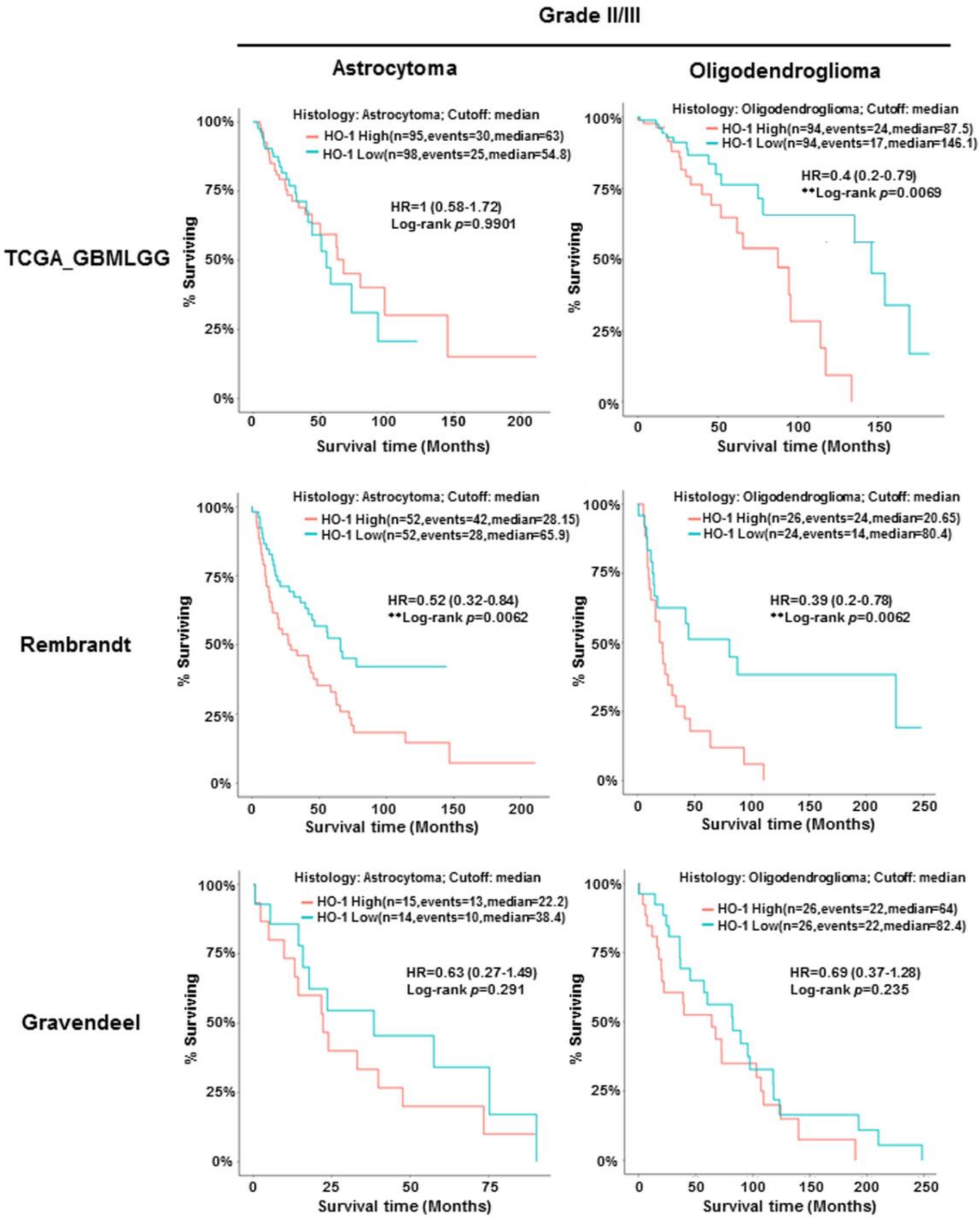

Supplementary Figure S7. Elevated *HO-1* gene expression is correlated with poorer survival in lower grade glioma patients

Kaplan-Meier survival analysis for human glioma Grade II/III (astrocytoma and oligodendroglioma) patients with higher and lower *HO-1* expression levels from TCGA_GBMLGG, Rembrandt and Gravendeel datasets. Higher and lower expression groups are divided by the median expression value under statistical analysis with the log-rank test.

Supplementary Table S1. Expression of 5-ALA metabolism enzyme genes in SP and MP cells

Gene Symbol	GenBank ID	Gene Name	SP	MP	Ratio (SP/MP)
Hmox1 (Ho-1)	NM_012580	heme oxygenase (decycling) 1	52.363	18.989	2.757
Alad	NM_012899	aminolevulinate dehydratase	33.470	43.883	0.763
Hmbs	NM_013168	hydroxymethylbilane synthase (porphobilinogen deaminase)	4.219	4.140	1.019
Uros	NM_001012068	uroporphyrinogen Ш synthase	0.174	0.185	0.944
Urod	NM_019209	uroporphyrinogen decarboxylase	20.168	20.571	0.894
Cpox	NM_001037095	coproporphyrinogen oxidase	0.706	0.747	0.946
Ppox	NM_001105968	protoporphyrinogen oxidase	0.687	0.761	0.902
Fech	NM_001108434	ferrochelatase	0.982	0.938	1.046
Blvra	NM_053850	biliverdin reductase A	0.987	0.860	1.147
Blvrb	NM_001106236	biliverdin reductase B (flavin reductase)	16.831	21.491	0.783
Ugt1a2	NM_201423	UDP-glucuronosyl transferase 1 family, polypeptide A2	0.783	0.738	1.061
Ugt1a6	NM_057105	UDP-glucuronosyl transferase 1 family, polypeptide A6	0.103	0.096	1.066
Alas1	NM_024484	aminolevulinate, delta-, synthase 1	26.466	16.984	1.558
Alas2	NM_013197	aminolevulinate, delta-, synthase 2	0.027	0.025	1.084

Supplementary Methods

Reagents

ABC transporter inhibitors reserpine and verapamil were purchased from Sigma (St Louis, MO, USA).

FACS analysis of PpIX with inhibitors of ABC transporters

The fluorescence intensity of cellular PpIX was determined by FACS. After SP and MP cells were sorted and re-cultured for 2 days, media were changed to fresh media containing 1 mM 5-ALA, and cells were additionally incubated for 4 hours. In some cases, 10 μM reserpine or 50 μM verapamil was added into media together with 5-ALA. PpIX fluorescence was measured with a FACS Aria II (BD Biosciences) using 488 nm laser and a 660/20 nm band-pass filter.

Cell viability assay

PpIX fluorescence low and high cells were sorted from cultured SP cells by FACS and collected by centrifugation. The cell suspension was prepared by a 1:1 dilution using a 0.4% Trypan blue solution and examined immediately under a microscope. The numbers of blue staining cells and total cells were counted.

Bioinformatic analysis

Gene expressions in different grades of gliomas and their correlation with

patients' clinical outcome were analyzed in TCGA_GBMLGG, Rembrandt and Gravendeel datasets by using GlioVis (http://gliovis.bioinfo.cnio.es/).

Statistical analysis

All comparisons between experimental groups, except for bioinformatics analysis, were made by Student's *t*-test. Target gene expression in different grade tumors was tested by pairwise *t* tests with corrections for multiple testing (*p* values with Bonferroni correction). For Kaplan-Meier survival curves, *p* values were obtained by using the log-rank test.
